# Introducing the Concept of the Minimally Important Difference to Determine a Clinically Relevant Change on Patient-Reported Outcome Measures in Patients with Intermittent Claudication

**DOI:** 10.1007/s00270-015-1060-0

**Published:** 2015-03-14

**Authors:** Anne P. Conijn, Wilma Jonkers, Ellen V. Rouwet, Anco C. Vahl, Jim A. Reekers, Mark J. W. Koelemay

**Affiliations:** 1 Departments of Vascular Surgery and Interventional Radiology, Academic Medical Center, Meibergdreef 9, 1105 AZ Amsterdam, The Netherlands; 2 Division of Health Care, Achmea Insurances, Burg. Roelenweg 13, 8021 EV Zwolle, The Netherlands; 3 Department of Vascular Surgery, Erasmus Medical Center, ‘s-Gravendijkwal 230, 3015 CE Rotterdam, The Netherlands; 4 Department of Vascular Surgery, Onze Lieve Vrouwe Gasthuis, Oosterpark 9, 1091 AC Amsterdam, The Netherlands; 5 Department of Radiology, Academic Medical Center, Meibergdreef 9, 1105 AZ Amsterdam, The Netherlands; 6 Department of vascular surgery, Academic Medical Center, Meibergdreef 9, 1105 AZ Amsterdam, The Netherlands

**Keywords:** Claudication, Clinical practice, Biostatistics

## Abstract

**Purpose:**

The minimally important difference (MID) represents the smallest change in score on patient-reported outcome measures that is relevant to patients. The aim of this study was to introduce the MID for the Vascular Quality of Life Questionnaire (VascuQol) and the walking impairment questionnaire (WIQ) for patients with intermittent claudication (IC).

**Methods:**

In this multicenter study, we recruited 294 patients with IC between July and October 2012. Patients completed the VascuQol, with scores ranging from 1 to 7 (worst to best), and the WIQ, with scores ranging from 0 to 1 (worst to best) at first visit and after 4 months follow-up. In addition, patients answered an anchor-question rating their health status compared to baseline, as being improved, unchanged, or deteriorated. The MID for improvement and deterioration was calculated by an anchor-based approach, and determined with the upper and lower limits of the 95 % confidence interval of the mean change of the group who had not changed according to the anchor-question.

**Results:**

For the MID analyses of the VascuQol and WIQ, 163 and 134 patients were included, respectively. The MID values for the VascuQol (mean baseline score 4.25) were 0.87 for improvement and 0.23 for deterioration. For the WIQ (mean baseline score 0.39), we found MID values of 0.11 and −0.03 for improvement and deterioration, respectively.

**Conclusion:**

In this study, we calculated the MID for the VascuQol and the WIQ. Applying these MID facilitates better interpretation of treatment outcomes and can help to set treatment goals for individual care.

**Electronic supplementary material:**

The online version of this article (doi:10.1007/s00270-015-1060-0) contains supplementary material, which is available to authorized users.

## Introduction

Since the treatment of patients with intermittent claudication (IC) is primarily aimed at improving their walking ability and health-related quality of life (HRQL), it is essential that these endpoints are measured when evaluating treatment. Walking ability is a part of a patient’s functional status (FS), and is frequently assessed using a treadmill test. However, treadmill tests do not correlate well with real-life walking distances, and are not an adequate reflection of the patient’s perceived walking impairment.[1, 2] Therefore, FS can better be assessed using patient-reported outcome measures (PROMs), such as the walking impairment questionnaire (WIQ) [3]. HRQL can be defined as the aspects of quality of life that relate specifically to a person’s health [4]. The Vascular Quality of Life Questionnaire (VascuQol) is an example of a disease-specific HRQL PROM for patients with peripheral artery disease (PAD) [5].

The importance of PROMs to evaluate treatment outcomes has been recognized by the vascular community, and they are used as endpoints in many clinical trials [6–9]. The next step will be to use PROMs in routine clinical practice. However, the interpretation of changes in PROM scores may be difficult when it is unknown how much change is actually considered relevant by patients. A statistically significant mean change in score after treatment in a sample doesn’t necessarily imply that an individual patient experiences a clinically meaningful change in his or her HRQL or FS.

The minimally important difference (MID) represents ‘the smallest change in score in the construct to be measured which patients perceive as important’ [10]. The MID can aid to better appreciate trial results and individual treatment results, can be calculated for all available PROMs and is relevant in all patient populations. This is illustrated in the following example. In a (fictional) clinical trial a PROM is used with a score range from 0 to 100. A statistically significant change in mean score for the patient sample from 25 to 33 was found, but it is unknown if this change is *relevant* to an individual patient. If, however, the MID for that PROM was known to be +10 points on the scale, it would be immediately clear that an individual patient would have to improve from a baseline score of 25 to at least 35 for the improvement to be clinically relevant.

The current study aims to introduce the concept of the MID for the VascuQol and the WIQ in patients with IC. This study was specifically not aimed at determining the effect of different treatment modalities.

## Methods

### Patients

The institutional review board (IRB) of the Academic Medical Center decided that this study met the criteria for exemption from IRB approval.

We used the patient sample of a prospective pilot study to determine the feasibility of PROMs as indicators of quality of care for patients with PAD. This study was conducted in cooperation with a Dutch health insurance company.

Patients were enrolled from July 2012 until October 2012 in nine hospitals in the Netherlands. Patients were eligible if they presented at the vascular surgery outpatient clinic with complaints of IC due to PAD and if they had not visited the outpatient clinic for symptomatic PAD in the previous year. Other inclusion criteria were sufficient knowledge of the Dutch language, an independent living situation, absence of psychiatric disorders, and the ability to communicate with the researchers.

### Treatment

As recommended by national guidelines, first line treatment was supervised exercise therapy (SET) in most patients [11]. Depending on physician and patient preferences percutaneous transluminal angioplasty (PTA) was sometimes used as primary treatment, and a few patients were treated with surgical revascularization. SET is only reimbursed by the Dutch health insurance when patients have additional insurance. Therefore in some patients treatment consisted of optimal medical therapy (OMT) (antiplatelet drug and a statin, advice to walk, and change lifestyle).

### Data Collection

In each centre, a local investigator was responsible for the execution of the study and data collection at baseline and at 3–4 months follow-up. Patient characteristics and questionnaires were sent to an independent trusted party (ITP) for further data linking and processing.

Data on smoking history, diabetes, pulmonary and cardiac diseases, renal function, previous vascular interventions (PTA or surgery), ankle brachial index (ABI), and number of affected legs were recorded at first visit in a pre-specified database. At follow-up, it was also recorded if a patient had received OMT or SET. No data on age and gender were recorded in this database, since these were retrieved when the ITP linked treatment codes (conservative, PTA or surgery) in the Dutch insurance billing system to patients in each hospital’s patient administration. However, because unblinding was impossible due to privacy reasons, it was impossible for the ITP to retrieve data on age and gender if no treatment code was listed. If no treatment code was listed, we used the data on treatment modality recorded by the local investigator at follow-up.

PROMs were handed out at the outpatient clinic or sent by mail. Patients returned the PROMs by mail to the local investigator. When necessary, patients were contacted by telephone to remind them and help fill in the PROM.

Only patients with available data on age and gender and a resting ABI <0.9 were analysed in the present study to ensure that the patient sample in the MID analysis had a proven diagnosis of IC due to PAD. Baseline characteristics and PROM scores of patients included and excluded from the MID analysis were compared.

### PROMs

The VascuQol is a disease-specific HRQL PROM, developed for patients with IC and critical limb ischemia [5]. It consists of five subscales (pain, symptoms, activities, emotional, and social) with 25 items in total. Each item is rated on a 7-point rating scale, with 1 representing the worst and 7 the best score. A total score, also ranging from 1 to 7, is calculated by dividing the sum of all items by 25. The VascuQol has been validated in Dutch [12, 13].

The WIQ is a PROM to rate walking impairment and consists of a speed, distance, and stairclimbing subscale with 14 items in total [3]. Patients rate their perceived difficulty of each item on a 5 point Likert scale. For example, patients are asked to assign a degree of difficulty with which they can walk 100 meters, with answers ranging from ‘no problems’ to ‘impossible’. Each item is weighted based on its difficulty. Subscale scores are calculated by adding the weighted scores, and dividing this by the maximum score so that each score ranges from 0 to 1, with lower scores indicating a higher level of impairment. An overall score is calculated as the mean of the three subscale scores. The WIQ has also been validated in Dutch [14, 15].

In addition to the PROMs, at follow-up patients filled in the following anchor-question: ‘Has your condition changed in the past three months?’ with the following response options: (a) improved, (b) unchanged, and (c) deteriorated.

### Imputation of Missing Items

Imputation of the VascuQol subscales took place if at least 50 % of the subscale was filled in. Missing values were imputed with the mean value of all the filled-in questions if this condition was satisfied, and under the assumption of “completely missing at random”.

When items were missing for the WIQ, we calculated a best- and worst-case scenario. We hereby took into account the questions the patients did fill in, and assumed that patients could never score higher on a harder task and never lower on an easier task. If the best- and worst-case scenario scores were no more than 0.25 points apart, we used the mean of these two values as the total WIQ score.

### Analysis

For the MID analysis, we used an anchor-based approach. Anchor-based approaches determine the MID by comparing PROMs to other measures or phenomena that have clinical relevance [16]. Revicki et al. suggested that the MID should be based on an anchor that has a correlation ≥0.3 with the PROM [17]. Therefore, Pearson correlation coefficients were calculated between the change in PROM scores and the anchor-question. The upper and lower limit of the 95 % confidence interval (CI) of the mean change of the group who indicated on the anchor-question that their situation had *not* changed after treatment represent the MID for improvement and deterioration, respectively.

Differences in baseline characteristics and PROM scores were determined with a student’s *t*-test for continuous variables, and with a Chi-square or Fisher’s exact test where appropriate for categorical variables. All analyses were performed using SAS enterprise guide version 5.1; SAS institute, Cary, NC, USA.

## Results

A total of 294 patients with IC were included in the pilot study. The VascuQol was sufficiently completed twice by 223 patients, the WIQ by 184 patients. After exclusion of patients with unknown age, gender, and resting ABI >0.9 there were 163 patients who were suitable for the MID analysis of the VascuQol, and 134 for the WIQ. Baseline characteristics of both the patients included and excluded from the analysis are shown in Table 1. All baseline characteristics and scores on PROMs were comparable for included and excluded patients, except for the ABI. Missing items for both PROMs are presented in Table S2 (online only).

**Table 1:** Baseline characteristics

Baseline characteristics	Total population (*n* *=* 294)	VascuQol (*n* *=* 163)	WIQ (*n* *=* 134)	Excluded patients VascuQol (*n* *=* 131)	Excluded patients WIQ (*n* *=* 160)	
Age (years, SD)	66.5 (± 10.4) (*n* *=* 271)	66.6 (±10.3)	65.9 (±10.1)	66.5 (±10.6) (*n* *=* 108)	67.1 (±10.7)(*n* *=* 137)	NS
Male/female gender	163/108 (*n* *=* 271)	95/68	76/58	68/40 (*n* *=* 108)	87/50 (*n* *=* 137)	NS
Current smoker	134 (45.9 %) (*n* *=* 292)	72 (44.2 %) (*n* *=* 162)	54 (40.3 %) (*n* *=* 129)	60 (48.4 %) (*n* *=* 124)	78 (48.8 %) (n = 152)	NS
History of smoking	271 (93.1 %) (n = 291)	141 (93.3 %) (n = 150)	123 (91.7 %) (n = 129)	97 (69.4 %) (n = 107)	115 (71.8 %) (*n* *=* 128)	NS
Diabetes	74 (25.2 %) (*n* *=* 284)	43 (26.3 %) (*n* *=* 161)	33 (24.6 %)	31 (23.6 %) (*n* *=* 123)	41 (25.6 %) (*n* *=* 150)	NS
Cardiac disease	74 (26 %) (*n* *=* 285)	43 (26.3 %) (*n* *=* 162)	35 (26.1 %)	31 (25.2 %) (*n* *=* 123)	39 (25.8 %) (*n* *=* 151)	NS
Lung disease	30 (10.2 %) (*n* *=* 84)	17 (10.4 %) (*n* *=* 162)	13 (9.7 %) (*n* *=* 134)	13 (9.9 %) (*n* *=* 122)	17 (10.6 %) (*n* *=* 150)	NS
eGFR						NS
<60	55 (19.3 %)	30 (18.4 %)	25 (18.6 %)	25 (19.1 %)	30 (18.6 %)	
>60	162 (55.1 %)	114 (69.9 %)	98 (73.1 %)	48 (36.6 %)	64 (40 %)	
Unknown	77	19	11	58	66	
Previous vascular intervention	97 (34.2 %) (*n* *=* 284)	51 (31.3 %) (*n* *=* 160)	40 (29.9 %) (*n* *=* 133)	46 (35.1 %) (*n* *=* 124)	57 (35.6 %) (*n* *=* 151)	
ABI at rest						*P* < 0.0001
<0.5	40 (13.6 %)	30 (18.4 %)	28 (20.9 %)	10 (7.6 %)	12 (7.5 %)	
0.5–0.75	112 (38.1 %)	83 (50.9 %)	71 (53 %)	29 (22.1 %)	41 (25.6 %)	
0.75–0.9	63 (21.4 %)	50 (30.7 %)	35 (26.1 %)	13 (9.9 %)	28 (17.5 %)	
0.9–1.1	18 (6.1 %)	0	0	18 (13.7 %)	18 (11.3 %)	
1.1–1.3	4 (1.4 %)	0	0	4 (3.1 %)	4 (2.5 %)	
unknown	57	0	0	57	57	
Affected legs						NS
Unilateral	125 (42.5 %)	78 (47.9 %)	68 (50.7 %)	47 (35.9 %)	57 (35.6 %)	
Bilateral	130 (44.2 %)	84 (51.5 %)	65 (48.5 %)	46 (35.1 %)	65 (40.6 %)	
Unknown	39	1	1	38	38	
Received treatment						
Conservative / optimal medical treatment	113	47	40	66	73	
SET	141	93	73	48	68	
Endovascular	17	10	9	7	8	
Surgical treatment	23	13	12	10	11	
Questionnaires						
Follow-up time (days, SD)		123 (27)	126 (26)			
Baseline VascuQol score		4.25 (1.2)		4.26 (*n* *=* 119)^a^		NS
Baseline WIQ score			0.39 (0.1)		0.42 (*n* *=* 120)^a^	NS

Data displayed as number (percentage) or mean (standard deviation)

^a^Score was calculated in the number of patients that did sufficiently fill in the baseline questionnaire, but were excluded for not sufficiently filling follow-up questionnaire

*NS* Not significant

### Calculation of the MID for the VascuQol

Table 2 shows that the mean improvement in VascuQol summary score was 0.83. The correlation between the anchor-question and the VascuQol was 0.47, thus meeting the criteria of Revicki [17].

**Table 2:** Distribution of scores and MID VascuQol

VascuQol (*n* *=* 163)			
Baseline	Follow up	Mean change score	Correlation with anchor-question
4.25 (1.20)	5.08 (1.28)	0.83	0.47

Anchor-question			
	*N*	Mean change on VascuQol	95 % CI
Improved	97	1.23	(1.01–1.46)
Unchanged	43	0.55	(0.23–0.87)
Deteriorated	23	−0.36	(−0.74 to 0.01)

MID VascuQol	
Improvement	0.87
Deterioration	0.23

The MIDs calculated by the anchor-based approach were 0.23 and 0.87, for deterioration and improvement, respectively (Table 2). This means that patients with an increase of ≥0.87 compared to their baseline score have improved in a clinically relevant way. For deterioration, we found an MID of 0.23. While one might expect a negative MID value for deterioration, the MID value found here indicates that an increase in VascuQol summary score of less than 0.23 points is actually experienced as deterioration by patients.

**Fig. 1:**
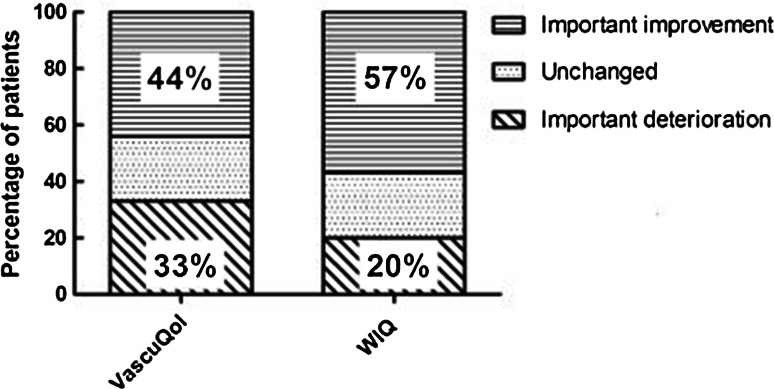
Proportion of patients that show a clinically relevant improvement and deterioration per PROM

Figure 1 shows the proportion of patients with a clinically relevant improvement or deterioration of their HRQL on the VascuQol. This figure shows that 44 % of the patients achieved a clinically meaningful improvement at follow-up. A clinically meaningful deterioration is seen in 33 % of the patients.

### Calculation of MID for the WIQ

Distribution of scores and details on MID calculation for the WIQ are presented in Table 3. The correlation between the anchor-question and the WIQ was 0.41, also meeting the criteria of Revicki [17].

**Table 3:** Distribution of scores and MID WIQ

WIQ (*n* *=* 134)			
Baseline	Follow up	Mean change score	Correlation with anchor-question
0.39 (0.24)	0.55 (0.28)	0.16	0.41

Anchor-question			
	N	Mean change on WIQ	95 % CI
Improved	79	0.25	(0.19–0.3)
Unchanged	37	0.04	(−0.03 to 0.11)
Deteriorated	18	0.01	(−0.05 to 0.06)

MID WIQ	
Improvement	0.11
Deterioration	–0.03

The MID values found were −0.03 and 0.11 for deterioration and improvement, respectively. Interpretation of the MIDs is similar to those of the VascuQol.

Figure 1 shows the proportion of patients that reached a clinically relevant improvement or deterioration on the WIQ. This figure shows that 57 % of the patients achieved a clinically meaningful improvement at follow-up. A clinically meaningful deterioration in walking impairment was seen in 20 %.

## Discussion

Outcomes that matter most to patients with IC are walking capacity and HRQL. These can be assessed using PROMs, which are common endpoints in trials, have the potential to support clinical management of patients and can help assess provider performance.

When interpreting changes in PROM scores there are some important points to consider. While physicians have a distinct idea which amount of change in clinical measures such as blood pressure is relevant, interpretation of PROM scores is less apparent. This is hampered even more by the fact that many PROMs have different rating scales (e.g., 0–1, 1–7, 1–100), making score changes incomparable. Furthermore, it is important to realize that in larger sample sizes the standard deviations of scores become smaller, resulting in earlier significant findings than in a small sample sizes. MID values indicate which amount of change is considered relevant by patients. They can be applied independent of sample size, and are thus useful in both individual care and research. In individual care, caregivers may decide to alter treatment strategy when after a certain period a patient doesn’t meet a relevant improvement. In research, a big advantage of applying MID values is that it helps display the proportion of patients in a sample that reaches a clinically relevant improvement. Concurrently, it can display how many patients show a clinically relevant deterioration despite treatment, as shown in Fig. 1. This would have been missed when only comparing the mean baseline score of the sample with the mean score after treatment, since this would have probably resulted in a positive mean change score, falsely indicating improvement for all patients in the sample. While it was beyond the scope of this paper, in future studies that compare treatment modalities it may be insightful to compare the proportion of patients that reach a clinically relevant improvement and deterioration per treatment group.

We found a positive MID-value for the VascuQol for deterioration. There are several explanations. It may be attributed to a learning effect, i.e., patients who do not improve (unchanged group) may still learn to fill in a PROM more accurately by repetition, resulting in a higher follow-up score, and thus a positive MID for deterioration. Furthermore, the VascuQol is a disease-specific PROM, in contrast to the anchor-question. Other conditions besides claudication may prevail when patients rate their overall condition. The VascuQol only takes into account the PAD-related problems. Therefore, the mean PROM score may increase, while the anchor-question is rated as unchanged.

MID values can be calculated for any PROM in any patient population. Many different methods for calculation exist. An overview can be found in the paper by Crosby et al. [16] Generally, calculation methods are divided into anchor-based approaches and distribution-based approaches. Anchor-based approaches determine the MID by comparing PROMs to other measures or phenomena that have clinical relevance. This can for example be an anchor-question, as we have shown in this study. Distribution-based approaches are based on statistical characteristics of the PROM scores in a patient sample. While studies have shown that values found in anchor-based and distribution-based approaches are often comparable, in calculations based on distribution-based approaches it is still not taken into account which amount of change is considered relevant by patients. Therefore, anchor-based approaches are always preferred.

Our study has some limitations. First, the proportion of patients that did not sufficiently complete the PROMs twice was substantial. This is a well-known problem and not exclusive to our study, but it should be considered when applying PROMs, since it limits their overall use. Second, to ensure that the study population was representative for all IC patients, we intentionally excluded patients of unknown age, gender, and/or ABI, which may have induced bias. Yet, the included and excluded patients did not differ in terms of baseline characteristics and PROM scores, and despite excluding many patients an acceptable sample was left for the MID analysis. Finally, we do not know how many patients refused to participate in the pilot study, and how this may have influenced MID values. Further studies are required to overcome these potential biases.

## Conclusion

We have calculated the MID values for two frequently used PROMs for patients with IC. As demonstrated in this study, the MID is a helpful tool to interpret the clinical relevance of changes in PROM scores, which may be used in research and individual care.

## Electronic supplementary material

Below is the link to the electronic supplementary material.

Supplementary material 1 (DOC 46 kb)
