# Supplementary material for: Introducing the Concept of the Minimally Important Difference to Determine a Clinically Relevant Change on Patient-Reported Outcome Measures in Patients with Intermittent Claudication
Source: Cardiovasc Intervent Radiol. 2015 Mar 14;38(5):1112–8. doi: 10.1007/s00270-015-1060-0 (PMC4565858; doi:10.1007/s00270-015-1060-0)
Supplement: Supplementary file 1 — Supplementary material 1 (DOC 46 kb) [file 270_2015_1060_MOESM1_ESM.doc]

Table S2. Missing items

| **VascuQol** | | | |
| --- | --- | --- | --- |
| No of excluded patients for MID analysis | | | 131 of 294 |
| **Missing items for included patients** | | | |
| Completed questions baseline | Completed questions follow-up | % missing | No of patients |
| 25 | 25 | 0% | 113(69%) |
| 24 | 25 | 2% | 19 (12%) |
| 25 | 24 | 2% | 10 (6%) |
| 23 | 25 | 4% | 3 (2%) |
| 24 | 24 | 4% | 4 (2%) |
| 25 | 23 | 4% | 4 (2%) |
| 22 | 25 | 6% | 2 (1%) |
| 23 | 24 | 6% | 2 (1%) |
| 24 | 23 | 6% | 2 (1%) |
| 25 | 21 | 8% | 1 (1%) |
| 22 | 23 | 10% | 1 (1%) |
| 24 | 19 | 14% | 1 (1%) |
| 18 | 22 | 20% | 1 (1%) |
| **WIQ** | | | |
| No of excluded patients for MID analysis | | | 160 of 294 |
| **Missing items for included patients** | | | |
| Baseline | Follow up | Δ max | No of patients |
| No missing items | No missing items | 0 | 100 (75%) |
| Unambiguous score | Unambiguous score | 0 | 9 (7%) |
| Unambiguous score | Max. difference 0.025 | 0.025 | 3 (2%) |
| Max difference 0.025 | Unambiguous score | 0.025 | 2 (1%) |
| Max difference 0.0625 | Unambiguous score | 0.0625 | 2 (1%) |
| Max difference 0.025 | Max difference 0.0625 | 0.0875 | 1 (1%) |
| Max difference 0.025 | Max difference 0.025 | 0.05 | 1 (1%) |
| Max difference 0.125 | Unambiguous score | 0.125 | 3 (2%) |
| Unambiguous score | Max difference 0.125 | 0.125 | 2 (1%) |
| Max difference 0.25 | Unambiguous score | 0.25 | 5 (4%) |
| Unambiguous score | Max difference 0.25 | 0.25 | 3 (2%) |
| Max difference 0.0625 | Max difference 0.25 | 0.3125 | 2 (1%) |
| Max difference 0.125 | Max difference 0.25 | 0.375 | 1 (1%) |

NB. For the WIQ, when items were missing, the maximum difference in score between the best and worst-case scenario is shown.
Where unambiguous score is noted, items may be missing but the there is no difference between possible scores.
